# P108 and T109 on E2 Glycoprotein Domain I Are Critical for the Adaptation of Classical Swine Fever Virus to Rabbits but Not for Virulence in Pigs

**DOI:** 10.1128/JVI.01104-20

**Published:** 2020-08-17

**Authors:** Libao Xie, Yuying Han, Yuteng Ma, Mengqi Yuan, Weike Li, Lian-Feng Li, Miao Li, Yuan Sun, Yuzi Luo, Su Li, Shouping Hu, Yongfeng Li, Hua-Ji Qiu

**Affiliations:** a State Key Laboratory of Veterinary Biotechnology, Harbin Veterinary Research Institute, Chinese Academy of Agricultural Sciences, Harbin, China; b State Key Laboratory of Veterinary Etiological Biology, Lanzhou Veterinary Research Institute, Chinese Academy of Agricultural Sciences, Lanzhou, China; Instituto de Biotecnologia/UNAM

**Keywords:** adaptation, classical swine fever virus, entry, virulence

## Abstract

The classical swine fever virus (CSFV) live attenuated vaccine C-strain is adaptive to rabbits and attenuated in pigs, in contrast with the highly virulent CSFV Shimen strain. Previously, we demonstrated that P108 and T109 on the E2 glycoprotein (E2^P108-T109^) in domain I (E2^DomainI^) rather than R132, S133, and D191 in domain II (E2^DomainII^) determine C-strain’s adaptation to rabbits (ATR) (Y. Li, L. Xie, L. Zhang, X. Wang, C. Li, et al., Virology 519:197–206, 2018). However, it remains elusive whether these critical amino acids affect the ATR of the Shimen strain and virulence in pigs. In this study, three chimeric viruses harboring E2^P108-T109^, E2^DomainI^, or E2^DomainII^ of C-strain based on the non-rabbit-adaptive Shimen mutant vSM-HCLVE^rns^ carrying the E^rns^ glycoprotein of C-strain were generated and evaluated. We found that E2^P108-T109^ or E2^DomainI^ but not E2^DomainII^ of C-strain renders vSM-HCLVE^rns^ adaptive to rabbits, suggesting that E2^P108-T109^ in combination with the E^rns^ glycoprotein (E2^P108-T109^-E^rns^) confers ATR on the Shimen strain, creating new rabbit-adaptive CSFVs. Mechanistically, E2^P108-T109^-E^rns^ of C-strain mediates viral entry during infection in rabbit spleen lymphocytes, which are target cells of C-strain. Notably, pig experiments showed that E2^P108-T109^-E^rns^ of C-strain does not affect virulence compared with the Shimen strain. Conversely, the substitution of E2^DomainII^ and E^rns^ of C-strain attenuates the Shimen strain in pigs, indicating that the molecular basis of the CSFV ATR and that of virulence in pigs do not overlap. Our findings provide new insights into the mechanism of adaptation of CSFV to rabbits and the molecular basis of CSFV adaptation and attenuation.

**IMPORTANCE** Historically, live attenuated vaccines produced by blind passage usually undergo adaptation in cell cultures or nonsusceptible hosts and attenuation in natural hosts, with a classical example being the classical swine fever virus (CSFV) lapinized vaccine C-strain, which was developed by hundreds of passages in rabbits. However, the mechanism of viral adaptation to nonsusceptible hosts and the molecular basis for viral adaptation and attenuation remain largely unknown. In this study, we demonstrated that P108 and T109 on the E2 glycoprotein together with the E^rns^ glycoprotein of the rabbit-adaptive C-strain confer adaptation to rabbits on the highly virulent CSFV Shimen strain by affecting viral entry during infection but do not attenuate the Shimen strain in pigs. Our results provide vital information on the different molecular bases of CSFV adaptation to rabbits and attenuation in pigs.

## Abstract (precis)

Historically, live attenuated vaccines produced by blind passage usually undergo adaptation in cell cultures or nonsusceptible hosts and attenuation in natural hosts, with a classical example being the classical swine fever virus (CSFV) lapinized vaccine C-strain, which was developed by hundreds of passages in rabbits. However, the mechanism of viral adaptation to nonsusceptible hosts and the molecular basis for viral adaptation and attenuation remain largely unknown. In this study, we demonstrated that P108 and T109 on the E2 glycoprotein together with the E^rns^ glycoprotein of the rabbit-adaptive C-strain confer adaptation to rabbits on the highly virulent CSFV Shimen strain by affecting viral entry during infection but do not attenuate the Shimen strain in pigs. Our results provide vital information on the different molecular bases of CSFV adaptation to rabbits and attenuation in pigs.

## INTRODUCTION

Viruses have a predefined host spectrum (1). However, viruses can breach the interspecies barrier through serial passaging in a nonsusceptible host, leading to viral adaptation to nonsusceptible hosts and attenuation in primary hosts. Well-known examples of this are the Chinese hog cholera lapinized virus (HCLV, also known as C-strain) and the lapinized rinderpest virus (2, 3). Remarkably, the viral infection is limited if one stage of the life cycle is blocked in nonsusceptible hosts, especially if the virus is unable to utilize a factor(s) which is necessary for infection or to evade a restriction factor(s). Hence, mechanisms of viral adaptation to nonsusceptible hosts by serial passaging are as follows: (i) improving entry efficiency (4, 5), (ii) enhancing the ability to antagonize or evade the nonsusceptible host's restriction factor(s) (6–8), and (iii) promoting the assembly and release of virus particles (9). Entry is the first and vital step during viral infection, which is mediated by the interaction between viral envelope proteins and cellular receptors (10).

Classical swine fever (CSF) is a devastating infectious disease of pigs caused by classical swine fever virus (CSFV), which often leads to huge economic losses to the pork industry (11). CSFV is a small, enveloped virus with a single-stranded positive RNA molecule encoding four structural proteins (C, E^rns^, E1, and E2) and eight nonstructural proteins (N^pro^, p7, NS2, NS3, NS4A, NS4B, NS5A, and NS5B) (12). The E^rns^ glycoprotein mediates viral attachment by interacting with LamR (13). The E2 glycoprotein, which forms homodimers and heterodimers with E1, is essential for viral entry into cells (14, 15) and also plays a pivotal role in viral tropism (16). Meanwhile, E^rns^ and E2 glycoproteins of CSFV are closely associated with virulence and pathogenicity in pigs (17–22).

In general, CSFV infects only domestic pigs and wild boar (23). However, the species barrier of CSFV between pigs and rabbits was crossed by hundreds of passages of a highly virulent strain in rabbits. C-strain, a live attenuated vaccine against CSF, was developed by passaging a highly virulent CSFV strain in rabbits in the 1950s and is characterized by adaptation to rabbits (ATR) and attenuation in pigs (3). Until now, however, the mechanism of the C-strain ATR and the molecular basis for ATR and attenuation in pigs have remained elusive.

Our previous study found that vSM-HCLVE^rns^ carrying the E^rns^ of C-strain in the background of the highly virulent CSFV Shimen strain is not adaptive to rabbits, indicating that the E^rns^ of C-strain alone could not render the Shimen strain adaptive to rabbits (24). In contrast, the E2 and E^rns^ glycoproteins of C-strain together are sufficient for enabling the adaptation of the Shimen strain to rabbits. Furthermore, we observed that the C-strain backbone chimeras harboring E2 domain I (E2^DomainI^) or crucial mutation sites (E2^P108L-T109I^) on E2^DomainI^ of the Shimen strain do not adapt to rabbits, while the chimera harboring E2 domain II (E2^DomainII^) of the Shimen strain is adaptive to rabbits (24). Therefore, P108 and T109 on E2^DomainI^ (E2^P108-T109^) of C-strain are responsible for its ATR, whereas E2^DomainII^ containing three different residues (R132, S133, and D191) of C-strain are not determinants of its ATR. Because of the above findings, we further investigated the effects of these critical amino acids of C-strain on the Shimen strain ATR and the virulence in pigs.

In the present study, we demonstrated that E2^P108-T109^ in combination with the E^rns^ glycoprotein (E2^P108-T109^-E^rns^) of C-strain confers adaptation to rabbits on the Shimen strain. Remarkably, our data revealed that E2^P108-T109^-E^rns^ of C-strain promotes viral entry during infection in the target cells using a series of pseudotyped viruses. Finally, we demonstrated that E2^P108-T109^-E^rns^ of C-strain, responsible for the CSFV ATR, does not affect viral virulence in pigs, whereas E2^DomainII^-E^rns^, which is not associated with the adaptation, attenuates the Shimen strain in pigs.

## RESULTS

### *In vitro* rescue and evaluation of chimeric viruses in the background of the Shimen strain.

**FIG 1:**
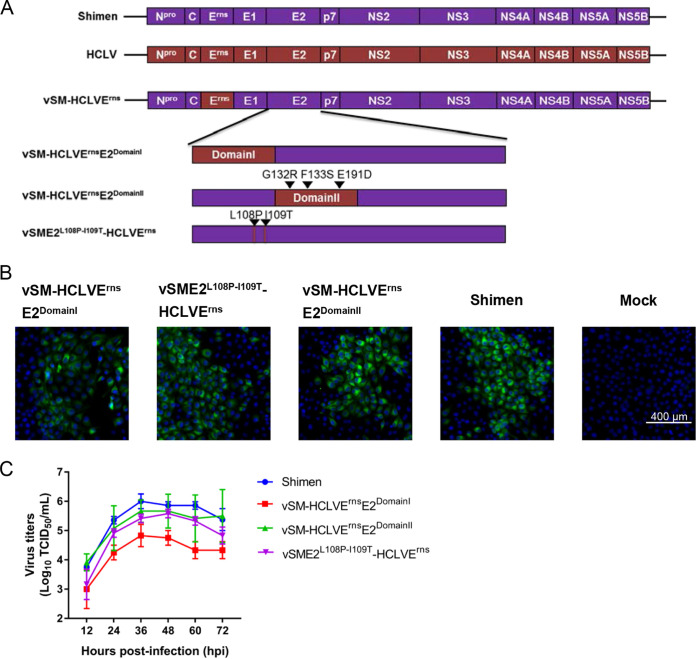
Generation and characterization of chimeric CSFVs. (A) Schematic diagram of the genomic organization of parental and chimeric viruses. Purple boxes indicate genes from the highly virulent Shimen strain, while red boxes indicate genes derived from the lapinized vaccine C-strain (also known as HCLV). The infectious clones of chimeric viruses (vSM-HCLVE^rns^E2^DomainI^, vSM-HCLVE^rns^E2^DomainII^, and vSME2^L108P-I109T^-HCLVE^rns^) were developed based on the infectious clone of the non-rabbit-adaptive chimeric virus vSM-HCLVE^rns^ carrying the E^rns^ glycoprotein of C-strain. (B) Indirect immunofluorescence staining of the PK-15 cells infected by chimeric viruses and parental virus. The nuclei were stained with 4′,6-diamidino-2-phenylindole (DAPI). Bar, 400 μm. (C) Growth kinetics of parental and chimeric viruses in PK-15 cells. The error bars represent the standard deviations for three replicates.

Based on previous results (24), we speculated that E2^P108-T109^ or E2^DomainI^ but not E2^DomainII^ of C-strain is necessary for conferring adaptation to rabbits on the non-rabbit-adaptive Shimen mutant vSM-HCLVE^rns^. Since vSM-HCLVE^rns^E2, harboring the E^rns^ and E2 glycoproteins of C-strain, showed better adaptation to rabbits than vSM-HCLVE1E2, containing the E1 and E2 glycoproteins of C-strain, according to the reproductive animal experiments in our previous study (24), three infectious clones (pSME2^L108P-I109T^-HCLVE^rns^, pSM-HCLVE^rns^E2^DomainI^, and pSM-HCLVE^rns^E2^DomainII^) were constructed based on the infectious clone pSM-HCLVE^rns^ containing E^rns^ of C-strain in the backbone of the Shimen strain (Fig. 1A). Chimeric viruses were rescued by transfecting individual infectious clones into swine kidney (SK6) cells, and transfected cells were serially passaged. Viral genomic sequencing results demonstrated that the sequences of the rescued chimeric viruses were as expected. The results of indirect immunofluorescence assay (IFA) demonstrated that the rescued viruses were infectious in the cells (Fig. 1B). The growth characteristics of the chimeras were determined in porcine kidney (PK-15) cells. Compared with the parental virus (Shimen strain), vSM-HCLVE^rns^E2^DomainI^ had lower viral titers at different time points, while viral titers of vSM-HCLVE^rns^E2^DomainII^ and vSME2^L108P-I109T^-HCLVE^rns^ were not significantly different (*P > *0.05) (Fig. 1C). Collectively, the expected chimeric viruses were generated.

### E2^P108-T109^-E^rns^ of C-strain confers adaptation to rabbits on the Shimen strain.

**FIG 2:**
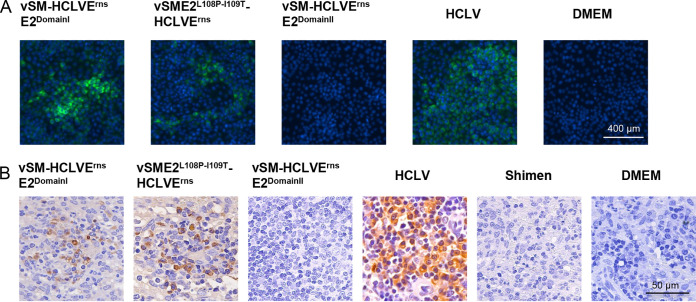
Rabbit-adaptive CSFV mutants were detected in rabbit spleens. (A) Indirect immunofluorescence staining of the SK6 cells infected with viruses isolated from the rabbit spleens and the nuclei stained with DAPI. Bar, 400 μm. (B) Viral antigens in the spleen samples detected by immunohistochemistry. The E2 glycoprotein was detected by anti-E2 antibody in the spleen lymphocytes from the rabbits inoculated with HCLV, vSM-HCLVE^rns^E2^DomainI^, or vSME2^L108P-I109T^-HCLVE^rns^ but not the Shimen strain, vSM-HCLVE^rns^E2^DomainII^, or DMEM. Bar, 50 μm.

To investigate whether E2^P108-T109^-E^rns^ of C-strain adapts the Shimen strain to rabbits, five groups of 24 rabbits were inoculated with different chimeric viruses (Table 1). Rectal temperature was recorded 24 h before and after inoculation and then every 6 h until 72 h postinoculation (hpi). According to the fever response standard described previously (24), vSME2^L108P-I109T^-HCLVE^rns^ and vSM-HCLVE^rns^E2^DomainII^ did not induce a fever response in rabbits, in contrast to C-strain (Table 1). Four rabbits from each group were randomly selected to be euthanized for determining copy numbers of the viral genome in the spleens by real-time reverse transcription-quantitative PCR (RT-qPCR). The viral genome was detected in the rabbits inoculated with vSME2^L108P-I109T^-HCLVE^rns^, vSM-HCLVE^rns^E2^DomainI^, or C-strain but not vSM-HCLVE^rns^E2^DomainII^ (Table 1). To further determine whether the progeny viruses were present in rabbit spleens, virus isolation was performed in SK6 cells. The IFA results demonstrated that the viruses were isolated from the spleens of rabbits inoculated with vSME2^L108P-I109T^-HCLVE^rns^, vSM-HCLVE^rns^E2^DomainI^, or C-strain but not with vSM-HCLVE^rns^E2^DomainII^ or Dulbecco’s modified Eagle’s medium (DMEM), and no mutation occurred, as confirmed by sequencing (Fig. 2A). The immunohistochemistry results showed that the E2 glycoprotein was expressed in the spleen lymphocytes of the rabbits inoculated with C-strain, vSM-HCLVE^rns^E2^DomainI^, or vSME2^L108P-I109T^-HCLVE^rns^ but not vSM-HCLVE^rns^E2^DomainII^ or the Shimen strain (Fig. 2B). These results indicated that chimeric viruses carrying E2^P108-T109^-E^rns^ or E2^DomainI^-E^rns^ of C-strain are adaptive to rabbits. Additionally, at 10 days postinoculation (dpi), anti-E2 antibodies were detected in the remaining two rabbits of each group (Table 1). As expected, we demonstrated that E2^P108-T109^ or E2^DomainI^ but not E2^DomainII^ of C-strain could render vSM-HCLVE^rns^ adaptive to rabbits, suggesting that E2^P108-T109^-E^rns^ of C-strain could confer adaptation to rabbits on the Shimen strain, generating new rabbit-adaptive CSFVs.

**TABLE 1:** Viral replication in the spleens of the rabbits inoculated with the Shimen-based chimeric viruses and HCLV

Inoculum	Dose (TCID_50_)	No. with fever/total	No. with viral replication/total	Mean viral RNA copies in the spleens (copies/μl)	No. with seroconversion at 10 dpi/total
vSME2^L108P-I109T^-HCLVE^rns^	10^4^	0/6	2/4	2.52 × 10^2^	2/2
vSM-HCLVE^rns^E2^DomainI^	10^4^	5/6	2/4	1.08 × 10^2^	2/2
vSM-HCLVE^rns^E2^DomainII^	10^4^	0/6	0/4	No count	2/2
HCLV	10^4^	4/4	2/2	6.30 × 10^3^	2/2
DMEM	1 ml	0/2	0/1	No count	0/1

### Growth curves of rabbit-adaptive CSFV mutants in primary rabbit spleen lymphocytes and swine macrophages.

**FIG 3:**
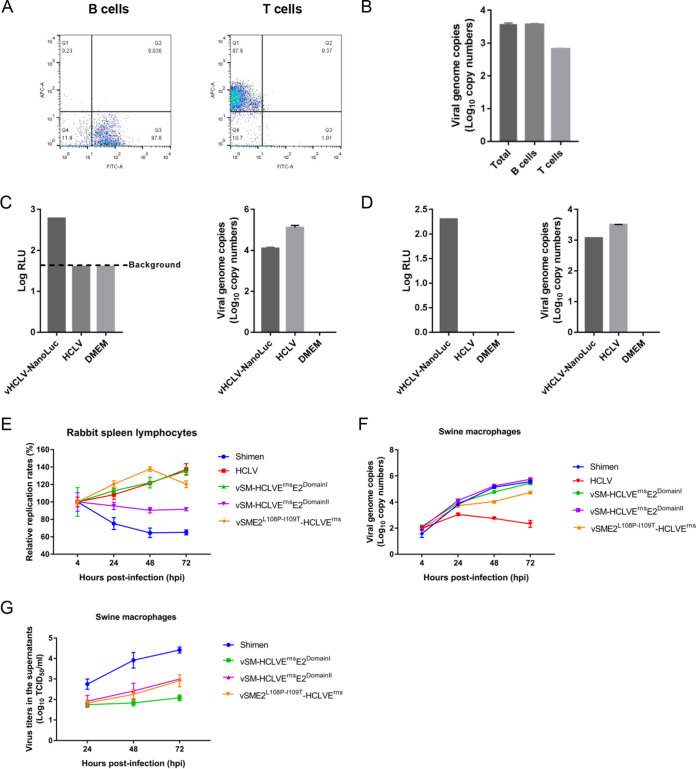
Growth curves of CSFV mutants in primary rabbit spleen lymphocytes and swine macrophages. (A) T cells and B cells isolated by flow cytometry. T cells and B cells from the spleen lymphocytes of rabbits inoculated with HCLV were isolated by flow cytometry. The cells in the Q1 region are T cells, and cells in the Q3 region are B cells. (B) Viral genome copy numbers in T cells and B cells isolated from rabbits inoculated with C-strain. The viral genome copy numbers in both T cells and B cells isolated by flow cytometry were determined using RT-qPCR. Total cells before sorting served as a control. (C) NanoLuc activities or viral genome copy numbers were tested in the spleen lymphocytes isolated from rabbits inoculated with vHCLV-NanoLuc, HCLV, or DMEM. (D) NanoLuc activities or viral genome copy numbers were tested in the spleen lymphocytes infected with vHCLV-NanoLuc or HCLV or treated with DMEM only. (E) Spleen lymphocytes isolated from healthy rabbits were infected with HCLV, the Shimen strain, vSM-HCLVE^rns^E2^DomainI^, vSM-HCLVE^rns^E2^DomainII^, or vSME2^L108P-I109T^-HCLVE^rns^ at an MOI of 0.01. Viral genome copy numbers were measured at 4, 24, 48, and 72 h postinoculation (hpi). Relative replication rates were analyzed based on the viral genome copy numbers at 4 hpi. (F) Viral genome copy numbers in primary swine macrophages infected with parental or chimeric viruses. (G) Titers of progeny viruses in the supernatants of primary swine macrophage cell cultures. The viral titers were determined in SK6 cells.

To examine the growth property of rabbit-adaptive CSFV mutants in rabbit cells, spleen lymphocytes were identified as target cells in the rabbits infected with C-strain based on the results of immunohistochemistry assay (Fig. 2B). To determine the exact cell type (T cells or B cells) in the spleens of the rabbits inoculated with C-strain, T cells and B cells were isolated from total spleen cells using the FACSAria cell-sorting system from rabbits inoculated with C-strain at 3 dpi (Fig. 3A). The viral genome copy numbers of C-strain in T cells and B cells were determined by RT-qPCR (Fig. 3B). Furthermore, viral genome copy numbers and NanoLuc activities were determined in spleen lymphocytes isolated from the rabbits inoculated with C-strain or the reporter virus vHCLV-NanoLuc (Fig. 3C). Importantly, luciferase activities were detected in the spleen lymphocytes infected with vHCLV-NanoLuc (Fig. 3D). These results indicated that C-strain infects the spleen lymphocytes (both T cells and B cells) of the rabbits.

Next, primary rabbit spleen lymphocytes were isolated and infected with the Shimen strain, C-strain, vSME2^L108P-I109T^-HCLVE^rns^, vSM-HCLVE^rns^E2^DomainI^, or vSM-HCLVE^rns^E2^DomainII^. The viral genome copy numbers in the cells were analyzed at different time points. The results showed that viral genome copy numbers of rabbit-adaptive CSFV mutants (vSME2^L108P-I109T^-HCLVE^rns^ and vSM-HCLVE^rns^E2^DomainI^) or the parental virus C-strain increased over time. In contrast, the viral genome copy numbers of non-rabbit-adaptive CSFVs (vSM-HCLVE^rns^E2^DomainII^ and the Shimen strain) decreased over time (Fig. 3E). Unfortunately, the progeny viruses in the supernatants were undetectable using IFA in SK6 cells due to low-level replication, which is consistent with observations in rabbits. These results further demonstrated that chimeric viruses vSME2^L108P-I109T^-HCLVE^rns^ and vSM-HCLVE^rns^E2^DomainI^ but not vSM-HCLVE^rns^E2^DomainII^ are adaptive to primary rabbit spleen lymphocytes.

Furthermore, primary swine macrophages were inoculated with a series of chimeric viruses. The viral genome copy numbers in macrophages or the titers of progeny viruses in the supernatants were determined at different time points. The viral genome copy numbers of the Shimen strain and three chimeric viruses were indistinguishable, except those of C-strain in primary swine macrophages (Fig. 3F). However, the viral titers in the supernatants of the macrophages infected with the Shimen strain were higher than those in cells infected with chimeric viruses (Fig. 3G).

### E2^P108-T109^-E^rns^ of C-strain affects viral entry in the target cells.

**FIG 4:**
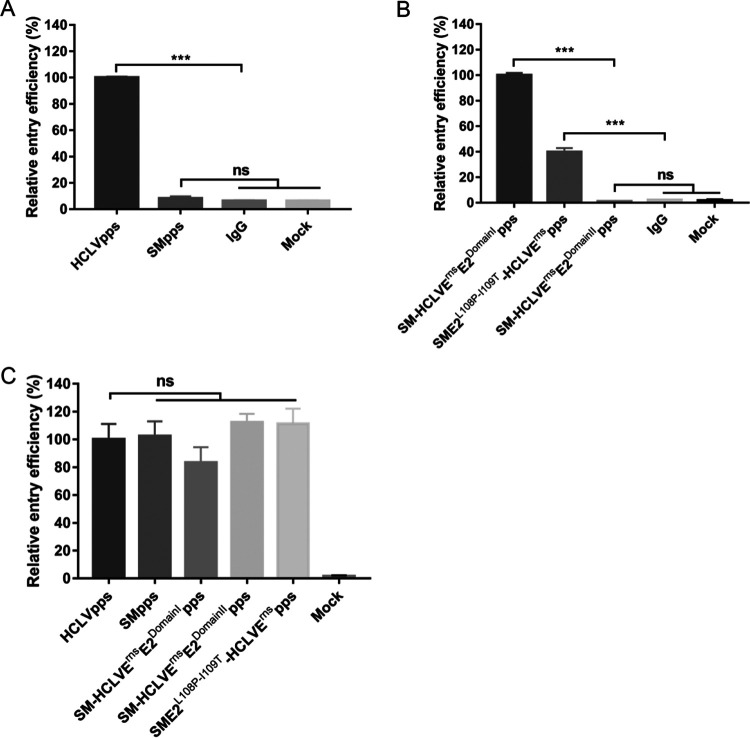
Entry was affected by the E2^P108-T109^-E^rns^ of C-strain. (A) HCLVpps but not SMpps could enter spleen lymphocytes of rabbits. Spleen lymphocytes were isolated from rabbits inoculated with 10^7^ transducing units (TU) of HCLVpps or SMpps or with 1 ml of DMEM. EGFP expression was detected in the spleen lymphocytes of the rabbits inoculated with HCLVpps but not SMpps or DMEM. (B) E2^P108-T109^-E^rns^ of C-strain affected viral entry into lymphocytes. Spleen lymphocytes were isolated from rabbits inoculated with 10^7^ TU of SME1-HCLVE^rns^E2^DomainI^pps, SME1-HCLVE^rns^E2^DomainII^pps, or SME1E2^L108P-I109T^-HCLVE^rns^pps or with 1 ml of DMEM. EGFP expression was detected in the spleen lymphocytes of the rabbits inoculated with SME1-HCLVE^rns^E2^DomainI^pps or SME1E2^L108P-I109T^-HCLVE^rns^pps but not those inoculated with SME1-HCLVE^rns^E2^DomainII^pps or DMEM. EGFP expression was detected using anti-EGFP antibody, and an irrelevant mouse IgG served as negative control. (C) The E2^P108-T109^-E^rns^ of C-strain did not affect viral entry into swine SK6 cells. EGFP expression was measured in SK6 cells infected with parental and chimeric pseudotyped viruses at an MOI of 2 at 48 hpi.

The E2 glycoprotein has been reported to be responsible for viral entry and tropism (14–16), while RNA replication is mediated by nonstructural proteins (NS3, NS4A-4B, and NS5A-5B) (25). Therefore, we speculated that the distinct adaptation potential of these chimeric viruses in rabbits may be due to different viral entry efficiency. A series of pseudotyped viruses (pps) bearing the E^rns^, E1, and E2 glycoproteins from the Shimen strain (SMpps) or C-strain (HCLVpps) were generated to investigate whether their differences occur in viral entry. We infected rabbits with SMpps or HCLVpps and isolated the spleen lymphocytes at 48 hpi. To amplify the fluorescence signals, an anti-EGFP (enhanced green fluorescent protein) antibody was used to measure EGFP expression. Meanwhile, mouse IgG was used to exclude nonspecific signals. We detected EGFP in the spleen lymphocytes from the rabbits inoculated with HCLVpps but not SMpps (Fig. 4A), suggesting that the C-strain ATR results from improved entry efficiency.

We further investigated the role of the E2^P108-T109^-E^rns^ of C-strain in viral entry using the chimeric pseudotyped viruses SME1-HCLVE^rns^E2^DomainI^pps, SME1-HCLVE^rns^E2^DomainII^pps, and SME1E2^L108P-I109T^-HCLVE^rns^pps. The results showed that EGFP was detected in the spleen lymphocytes from rabbits inoculated with SME1-HCLVE^rns^E2^DomainI^pps or SME1E2^L108P-I109T^-HCLVE^rns^pps but not in those from SME1-HCLVE^rns^E2^DomainII^pps-inoculated animals (Fig. 4B), indicating that E2^P108-T109^-E^rns^ of C-strain plays decisive roles in viral entry. Meanwhile, these pseudotyped viruses could enter swine SK6 cells with similar efficiency (Fig. 4C).

### E2^P108-T109^-E^rns^ of C-strain does not attenuate the Shimen strain in pigs.

**FIG 5:**
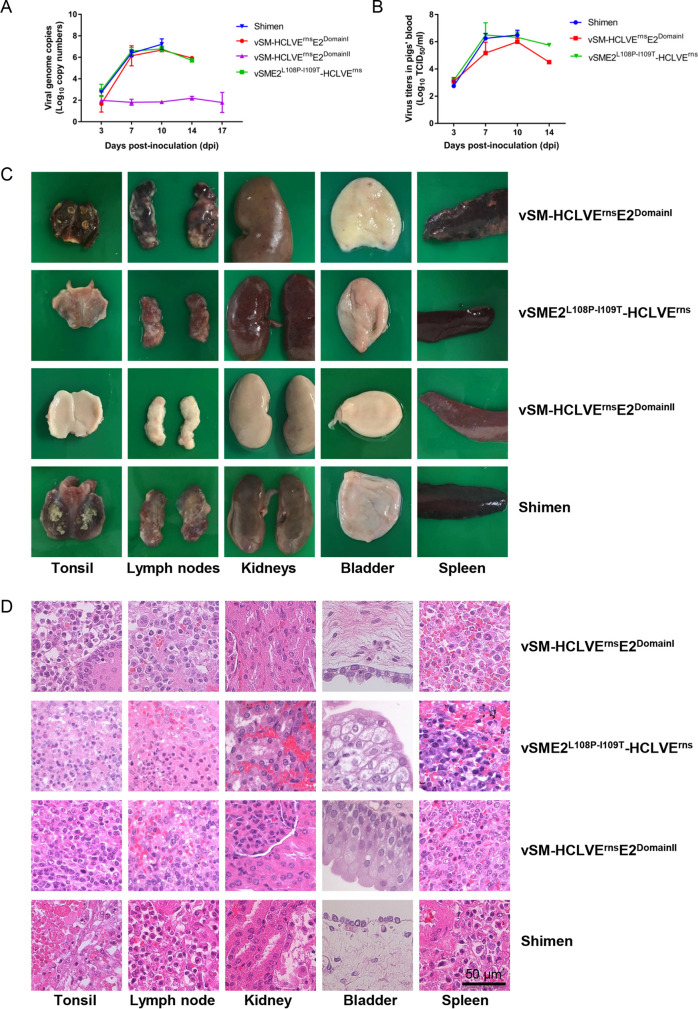
Viremia and representative pathological and histopathological changes of the pigs inoculated with chimeric viruses. (A) Viral genome copy numbers in the blood samples of the pigs inoculated with vSME2^L108P-I109T^-HCLVE^rns^, vSM-HCLVE^rns^E2^DomainI^, vSM-HCLVE^rns^E2^DomainII^, or the Shimen strain. (B) Viral titers in the blood samples from the pigs inoculated with vSM-HCLVE^rns^E2^DomainI^, vSME2^L108P-I109T^-HCLVE^rns^, or the Shimen strain in SK6 cells. (C) Representative pathological changes of various organs from the inoculated pigs. (D) Histopathological changes of various organs from the inoculated pigs. Bar, 50 μm.

To examine whether the key amino acids contributing to viral ATR affect virulence in pigs, four groups of pigs were inoculated intramuscularly (i.m.) with vSME2^L108P-I109T^-HCLVE^rns^, vSM-HCLVE^rns^E2^DomainI^, vSM-HCLVE^rns^E2^DomainII^, and the Shimen strain, using 10^5^ 50% tissue culture infective doses (TCID_50_). Clinical signs and rectal temperatures were monitored. All the pigs showed typical clinical signs (26) of CSF starting at 3 to 5 dpi, and the pigs inoculated with vSME2^L108P-I109T^-HCLVE^rns^, vSM-HCLVE^rns^E2^DomainI^, or the Shimen strain died at 11 to 16 dpi (Table 2). However, the temperatures of the pigs inoculated with vSM-HCLVE^rns^E2^DomainII^ normalized at 11 dpi, and the pigs survived until the end of this experiment. Viremia kinetics in animals inoculated with vSME2^L108P-I109T^-HCLVE^rns^ or vSM-HCLVE^rns^E2^DomainI^ were indistinguishable from those induced by the parental Shimen strain (Fig. 5A and B). In contrast, the pigs inoculated with vSM-HCLVE^rns^E2^DomainII^ showed lower viremia than the parental virus (Fig. 5A), while virus was undetectable in the blood samples from the pigs inoculated with vSM-HCLVE^rns^E2^DomainII^ using IFA in SK6 cells (Fig. 5B).

**TABLE 2:** Swine survival and fever response following inoculation with chimeric viruses and the parental Shimen strain

Viruses	No. of survivors/total	Mean time to death (days)^*a*^	Fever response^*a*^		
			No. of days to onset	Duration (days)	Maximum avg temp (°C)
vSME2^L108P-I109T^-HCLVE^rns^	0/3	13 (1.63)	3 (0)	9.3 (0.94)	41.7 (0.16)
vSM-HCLVE^rns^E2^DomainI^	0/3	13 (2.16)	3 (0)	10 (1.41)	41.8 (0.08)
vSM-HCLVE^rns^E2^DomainII^	3/3		2.67 (0.47)	6.7 (0.47)	41.1 (0.08)
Shimen	0/2	12.5 (1.5)	3 (0)	9.5 (1.5)	41.7 (0.05)

a Values are means (standard deviations).

The pigs inoculated with vSME2^L108P-I109T^-HCLVE^rns^, vSM-HCLVE^rns^E2^DomainI^, or the Shimen strain displayed CSF-specific pathological changes (including enlargement and hemorrhages in the lymph nodes, petechiae in the kidneys, and infarcts in the spleen) (25). In contrast, no obvious or slight pathological lesions were observed in the pigs inoculated with vSM-HCLVE^rns^E2^DomainII^ (Fig. 5C).

Moreover, histopathological examination of the various organs from the pigs inoculated with vSM-HCLVE^rns^E2^DomainII^ showed slight histopathological lesions, including depletion in lymph nodes and reactive hyperplasia in spleens. However, the pigs inoculated with vSM-HCLVE^rns^E2^DomainI^ or vSME2^L108P-I109T^-HCLVE^rns^ showed histopathological lesions similar to those in pigs inoculated with the Shimen strain in tonsils (lymphopenia), kidneys (denaturalization and necrosis in some tubular epithelia), and bladder (epithelial degeneration) (Fig. 5D).

Taken together, these observations indicate that E2^P108-T109^-E^rns^ of C-strain contributes to the ATR but does not alter the virulence of the Shimen strain in pigs, whereas E2^DomainII^-E^rns^ of C-strain attenuates the Shimen strain, suggesting that there are different molecular determinants of CSFV ATR and virulence in pigs.

## DISCUSSION

C-strain, which was developed by passaging a highly virulent CSFV strain in rabbits, is adaptive to rabbits and attenuated in pigs. It has been established that for the Shimen strain, acquiring the adaptation to rabbits depends on E2 together with E^rns^ or E1 of C-strain. Furthermore, P108 and T109 in E2^DomainI^ are crucial amino acids for C-strain to be adaptive to rabbits (24). In the present study, three chimeric viruses containing E2^P108-T109^, E2^DomainI^, or E2^DomainII^ of C-strain in the backbone of the non-rabbit-adaptive Shimen mutant vSM-HCLVE^rns^ were generated and evaluated for CSFV ATR and virulence in pigs. Our study shows for the first time that E2^P108-T109^ or E2^DomainI^ but not E2^DomainII^ of C-strain could render vSM-HCLVE^rns^ adaptive to rabbits, suggesting that E2^P108-T109^-E^rns^ of C-strain confers adaptation to rabbits on the Shimen strain. Importantly, we demonstrated that E2^P108-T109^-E^rns^ of C-strain mediated the adaptation of CSFV by prompting viral entry during infection in rabbit spleen lymphocytes. However, pathogenicity analysis in pigs showed that E2^P108-T109^-E^rns^ of C-strain did not alter the virulence of the Shimen strain in pigs.

It has been demonstrated that the E2 glycoproteins of pestiviruses are associated with viral tropism. A chimeric pestivirus containing border disease virus or CSFV E2 glycoprotein in the background of bovine viral diarrhea virus (BVDV) alters the tropism to different cells in contrast to the parental BVDV (13, 27). Recombinant E2 glycoproteins derived from three different pestiviruses have different abilities to modify BVDV and CSFV to be able to infect permissive cells, suggesting that the E2 glycoprotein is involved in host tropism of pestiviruses at the entry stage (28). Our findings demonstrated that E2^P108-T109^-E^rns^ of C-strain is responsible for the CSFV ATR, which further indicates the important roles of E2 and E^rns^ in the tropism of pestiviruses. However, the amount of replication that takes place in primary rabbit spleen lymphocytes is lower than that in primary swine macrophages. The distinct replication ability of CSFV in rabbits and pigs was observed. The possible explanations are as follows: the rabbit is not the natural host for CSFV, the adaptive ability of CSFV is low in primary rabbit spleen lymphocytes, and there is no cell-cell junction in suspended primary rabbit spleen lymphocytes.

The mechanism of viral adaptation to a heterogeneous host by passaging in nonsusceptible hosts can be associated with mutations in the viral genome, which can improve entry efficiency and promote viral genome replication and virion assembly and release (5, 29). Among these possible mechanisms, entry is the first and most important step for viral infection, and it is determined by the viral surface proteins. It has been reported that the highly conserved residue Q226 is associated with H2N2 and H3N2 viral adaptation to human receptors (4, 30). Three mutations in the viral glycoproteins E1 (L216R) and E2 (V388G and M405T) improve the efficiency of hepatitis C virus (HCV) entry into Lunet N mCD81 cells, which are likely to promote exposure of the CD81-binding site (5). Mutation of A281 was observed during HIV adaptation in macaques and affected the ability of the HIV-1 Env to use macaque CD4 (31). For pestiviruses, increasing evidence has proved that entry is mediated by several glycoproteins. The E^rns^ glycoprotein of pestiviruses attaches to the virion envelope by directly interacting with the E2 glycoprotein to form E^rns^-E2 heterodimers and is indispensable for virus attachment and infection of target cells (32). BVDV and CSFV belong to the genus *Pestivirus*, sharing similar properties (33), and E1-E2 heterodimers are essential for BVDV entry (34). In our study, E2^P108-T109^-E^rns^ of C-strain was demonstrated to be associated with the adaption of CSFV to rabbits by affecting viral entry during infection, which expands our knowledge of the entry of pestiviruses.

Typically, live attenuated vaccines developed by blind passage in cell cultures or nonsusceptible hosts are adaptive to the nonsusceptible host while being attenuated in the primary cell or host. For example, the adaptation of African swine fever virus to Vero cells leads to a gradual attenuation of virulence in pigs (35). Attenuation of the strain PC22A of porcine epidemic diarrhea virus was achieved by cell culture passage (36). Live attenuated vaccines such as C-strain and the lapinized rinderpest virus were developed by passaging a highly virulent strain in rabbits. However, the molecular basis for adaptation and attenuation remains largely unclear. Mutations in the surface glycoprotein E increase the adaptation of tick-borne encephalitis virus to BHK-21 cells and significantly attenuate neuroinvasiveness in adult mice (37). A single mutation in VP2 (A221T) confers the adaptation of infectious pancreatic necrosis virus to CHSE cells and attenuates virulence in Atlantic salmon fry (38). In contrast, D279N and A284T mutations can confer infectious bursal disease virus adaptation in cell culture but do not lead to virus attenuation (39). It was reported that the E2 glycoprotein of the CS vaccine strain derived from the LK VNIIVIM parental vaccine strain (40) could markedly attenuate the CSFV Brescia strain (18). Furthermore, many residues on the E2 were identified as being associated with CSFV virulence, including a discrete epitope (TAVSPTTLR) (19), W871T, W875D, and V878T in the internal fusion peptide (17), T830A (41), and T745I and M979K (22). The N269A/Q substitution, which removed the putative glycosylation site in the E^rns^ glycoprotein, decreased virulence of the Brescia strain (21). Notably, our results showed that E2^P108-T109^-E^rns^ of C-strain, which is associated with CSFV ATR, did not affect CSFV virulence in pigs. The E^rns^ glycoprotein of C-strain does not affect the virulence of the chimeric viruses, which may be due to the N269 present on both C-strain and the Shimen strain (21). However, E2 glycoprotein domain II, which is irrelevant to adaptation, alters virulence, further demonstrating that the molecular determinants of the ATR are different from those of attenuation of CSFV in pigs.

In summary, we demonstrated that E2^P108-T109^-E^rns^ of C-strain determines the adaptation of CSFV by facilitating viral entry during infection of rabbits. Furthermore, the molecular determinants of CSFV ATR do not affect virulence in pigs. Our findings contribute to our understanding of the molecular basis for adaptation and attenuation of live attenuated vaccines developed by blind passage in cell cultures or hosts. This study also implies that novel live attenuated vaccines against CSF may be developed by targeting genetic modifications instead of random evolution through blind cell passage.

## MATERIALS AND METHODS

### Cells and viruses.

SK6 and PK-15 cells were cultured with Dulbecco’s modified Eagle’s medium (DMEM) (catalog no. C11995500BT; Gibco) supplemented with 5% heat-inactivated fetal bovine serum (FBS) (catalog no. 10099-141C; Gibco) in a 37°C incubator with 5% CO_2_. HEK293T cells were cultured with DMEM supplemented with 10% FBS. The spleen lymphocytes of rabbits were maintained in Roswell Park Memorial Institute (RPMI) 1640 medium (catalog no. C11875500BT; Gibco) supplemented with 10% FBS, 1% antibiotics-antimycotics (catalog no. 15240-062; Gibco), 1% l-glutamine, and 0.20 ng/ml interleukin 2 (IL-2) (catalog no. ab119439; Abcam) (42). Primary swine macrophages were prepared as described previously (43). The CSFV C-strain (GenBank no. AY805221) and Shimen strain (GenBank no. AF092448.2) were used for the construction of infectious cDNA clones.

### Generation of chimeric viruses.

Based on the infectious cDNA clone pSM-HCLVE^rns^, which harbors E^rns^ of C-strain in the background of the Shimen strain (24), we took advantage of XhoI and BamHI restriction sites to construct pSM-HCLVE^rns^E2^DomainI^, pSM-HCLVE^rns^E2^DomainII^, and pSME2^L108P-I109T^-HCLVE^rns^ using fusion PCR with the primers listed in Table 3. The E2 domains I and II were amplified from pCSFV-HCLV using primers pSM-HCLVE^rns^E2^DomainI^-2F/2R and pSM-HCLVE^rns^E2^DomainII^-2F/2R, respectively. The PCR products were fused with products from pSM-HCLVE^rns^E2^DomainI^-1F/1R and pSM-HCLVE^rns^E2^DomainI^-3F/3R or from pSM-HCLVE^rns^E2^DomainII^-1F/1R and pSM-HCLVE^rns^E2^DomainII^-3F/3R. The PCR products obtained with the pSM-HCLVE^rns^ template using pSME2^L108P-I109T^-HCLVE^rns^-1F/1R and pSME2^L108P-I109T^-HCLVE^rns^-2F/2R primers designed for site-specific mutagenesis were fused with pSME2^L108P-I109T^-HCLVE^rns^-1F/2R. Both fusion PCR products and pSM-HCLVE^rns^ were digested with XhoI and BamHI and then linked with T4 DNA ligase (catalog no. M0202S; New England BioLabs). All these constructed infectious cDNA clones were identified by PCR, enzyme digestion, and sequencing.

**TABLE 3:** Primers used in this study

Primers	Sequences (5′–3′)
pSM-HCLVE^rns^E2^DomainI^-1F	CCACCTCGAGATGCTATGTGG
pSM-HCLVE^rns^E2^DomainI^-1R	GGAATGCAATGGTTGATGCGCTATTCCAGACCCTGGTTAA
pSM-HCLVE^rns^E2^DomainI^-2F	TTAACCAGGGTCTGGAATAGCGCATCAACCATTGCATTCC
pSM-HCLVE^rns^E2^DomainI^-2R	CTTCGGTTGATGGGTTGGTCCCGTCGAACAGGAGCTCGAATG
pSM-HCLVE^rns^E2^DomainI^-3F	CATTCGAGCTCCTGTTCGACGGGACCAACCCATCAACCGAAG
pSM-HCLVE^rns^E2^DomainI^-3R	TAGATGGATCCTCTCCACTAT
pSM-HCLVE^rns^E2^DomainII^-1F	CACCTCGAGATGCTATGTGGACG
pSM-HCLVE^rns^E2^DomainII^-1R	CCTCAGTTGATGGGTTGGTCCCGTCGAACAGGAGCTCGAATGTCACG
pSM-HCLVE^rns^E2^DomainII^-2F	CGTGACATTCGAGCTCCTGTTCGACGGGACCAACCCATCAACTGAGG
pSM-HCLVE^rns^E2^DomainII^-2R	CTTCATTTTCCACTGTGGTGGTCACACAATCCATTCTGTGCGG
pSM-HCLVE^rns^E2^DomainII^-3F	CCGCACAGAATGGATTGTGTGACCACCACAGTGGAAAATGAAG
pSM-HCLVE^rns^E2^DomainII^-3R	GATGGATCCTCTCCACTATAATAG
pSME2^L108P-I109T^-HCLVE^rns^-1F	CCACCTCGAGATGCTATGTGG
pSME2 ^L108P-I109T^-HCLVE^rns^-1R	CTCGAATGTCACGGAAGTGGGTAAAGCCCCCTTATGC
pSME2 ^L108P-I109T^-HCLVE^rns^-2F	GCATAAGGGGGCTTTACCCACTTCCGTGACATTCGAG
pSME2 ^L108P-I109T^-HCLVE^rns^-2R	TAGATGGATCCTCTCCACTAT

Three chimeric viruses were rescued as described previously with a slight modification (24). Six micrograms of each plasmid mixed with 6 μl of X-tremeGENE HP DNA transfection reagent (catalog no. 6366546001; Roche) was transfected into SK6 cells cultured in a 6-well plate. The transfected cells were passaged several times, and the supernatants were subjected to detection of the E^rns^ glycoprotein by a CSFV antigen test kit (catalog no. 99-40939; IDEXX). The positive samples were identified by RT-PCR, sequencing, and IFA.

### IFA and virus titration.

The viral titers in the rabbit spleens or the pig blood samples at different time points were determined as previously described (44). SK6 or PK-15 cells were inoculated with serial 10-fold dilutions of the samples and cultured in 37°C for 48 h. Cold absolute ethanol was used for fixing cells at −20°C for 20 min. The fixed cells were washed three times with phosphate-buffered saline (PBS) and incubated with an anti-E2 polyclonal antibody at 37°C for 2 h (45). After five washes with PBS, cells were incubated with Alexa Fluor 488 goat anti-rabbit IgG (catalog no. A11034; Roche) at 37°C for 1 h and then washed five times with PBS. Then, the cells were stained with 0.5 μg/ml 4′,6-diamidino-2-phenylindole (DAPI; catalog no. C0060; Solarbio) for 15 min and washed three times with PBS. The cells were analyzed for green fluorescence using an inverted fluorescence microscope (EVOS FL; Life Technologies). The viral titers were calculated according to the Reed-Muench method and expressed as median tissue culture infective doses (TCID_50_) per milliliter (46).

### Growth curves of the rescued viruses.

To determine the multistep growth curves of the rescued viruses, PK-15 cells cultured in 24-well plates were infected with these rescued viruses at a multiplicity of infection (MOI) of 0.1. Two hours later, the supernatants were removed, and the cells were washed three times with PBS. Then, fresh DMEM supplemented with 2% FBS was added to each well, and the cells were cultured at 37°C and 5% CO_2_. The cells were harvested at 12-h intervals and used to determine viral titers.

### RNA extraction and RT-qPCR.

Total RNA from tissues or cells was extracted using RNAiso Plus (catalog no. 9109; TaKaRa) according to the manufacturer’s protocol. cDNA synthesis was processed in a 20-μl volume with avian myeloblastosis virus (AMV) reverse transcriptase XL (catalog no. 2621; TaKaRa). The copy numbers of the CSFV genome were determined using a previously described RT-qPCR assay (47).

### Luciferase assay.

The reporter virus vHCLV-NanoLuc expressing the NanoLuc protein fused with the N^pro^ protein (between the amino acids 13 and 14) based on C-strain was generated. Rabbit spleen lymphocytes infected with vHCLV-NanoLuc or C-strain or treated with DMEM only were lysed with 100 μl of passive lysis buffer (catalog no. N1150; Promega) and incubated on a shaker for 1 h at 4°C. The supernatants were collected by centrifuging at 12,000 × *g* for 10 min at 4°C. NanoLuc activities were measured with EnVision multilabel plate readers (PerkinElmer).

### Flow cytometry.

The spleens from the rabbits inoculated with C-strain were collected after rabbits were euthanized at 3 dpi, and spleen cells were obtained by smearing the organs on 200-mesh copper wire mesh with RPMI 1640 medium. The red blood cells were lysed using red blood cell lysis buffer (catalog no. R1010; Solarbio), and the lymphocyte suspensions were washed with PBS. The following antibodies were used for flow cytometry, according to the manufacturers’ instructions: goat anti-rabbit IgM μ chain preadsorbed to secondary antibody (DyLight 488) (catalog no. ab98454; Abcam) for isolating B cells, mouse anti-rabbit T lymphocytes (catalog no. MCA800GA; Bio-Rad) for isolating T cells, and Alexa Fluor 633 goat anti-mouse IgG (heavy plus light chain [H+L]) (catalog no. A21052; Invitrogen) as the secondary antibody. Stained mononuclear cells from the spleens of the rabbits infected with C-strain were added to cytometry tubes and sorted with a high-speed cell sorter (MoFlo XDP; Beckman Coulter).

### Infection of primary cells from rabbits or pigs with chimeric viruses.

Primary rabbit spleen lymphocytes or swine macrophages were isolated from the animals and infected with parental or chimeric viruses with an MOI of 0.01. After 2 h of incubation in a CO_2_ incubator, the infected cells were washed three times with PBS and further incubated for 4, 24, 48, or 72 h (17). The viral genome copy numbers or the viral titers at different time points were measured.

### Preparation of pseudotyped viruses.

The DNA fragments encoding the last 60 amino acids of the C, E^rns^, E1, and E2 proteins from the infectious clones of C-strain, the Shimen strain, pSME1-HCLVE^rns^E2^DomainI^, pSME1-HCLVE^rns^E2^DomainII^, and pSME1E2^L108P-I109T^-HCLVE^rns^ were amplified and inserted into the pCAGGS vector (15, 48). Pseudotyped viruses were packaged by cotransfection into HEK293T cells with pNL4.3-GFP-ΔEnv and pCAGGS-HCLVE^rns^E1E2, pCAGGS-SME^rns^E1E2, pCAGGS-SME1-HCLVE^rns^E2^DomainI^, pCAGGS-SME1-HCLVE^rns^E2^DomainII^, or pCAGGS-SME1E2^L108P-I109T^-HCLVE^rns^ (49). At 48 h posttransfection (hpt), the supernatants were collected and centrifuged for concentration using Amicon Ultra centrifugal filters (catalog no. UFC901096; Amicon). HIV p24 antigen content was assessed by enzyme-linked immunoassay (ELISA) (catalog no. BF06203; Biodragon Immunotechnologies).

### Experimental infection of rabbits with chimeric viruses.

Twenty-four 14-week-old New Zealand White rabbits were divided into 5 groups and inoculated intravenously (i.v.) via the marginal ear vein with the viruses indicated in Table 1. The rectal temperature of all rabbits was monitored every 6 h from 24 to 72 hpi as described previously (24). Four rabbits were selected randomly from each group and euthanized at 3 hpi. The viral genome copy numbers in the spleens of the rabbits were determined and virus isolation was performed as described previously (24). The anti-E2 antibodies of the remainder rabbits were tested at 10 hpi using the classical swine fever virus antibody test kit (catalog no. 99-43220; IDEXX) according to the manufacturer's manuals.

### Experimental infection of rabbits with pseudotyped viruses.

The rabbits were inoculated i.v. with different chimeric pseudotyped viruses. Spleen lymphocytes were isolated at 48 hpi. Mouse anti-coral green fluorescent protein (cGFP)-tagged monoclonal antibody (MAb) (catalog no. A00185; GenScript) was used to detect the expression of EGFP in lymphocytes, and irrelevant mouse IgG (catalog no. A7028; Beyotime) was used as a negative control. As the secondary immunoreagent, fluorescein isothiocyanate-labeled goat anti-mouse IgG (catalog no. A11029; Invitrogen) was used. All antibodies mentioned were diluted at 1:200 in PBS. For flow cytometry analysis, 10^6^ cells in each sample were permeabilized with 0.15% Triton X-100. The cells were further incubated with primary antibody for 1 h and washed three times for 5 min with PBS. Then the cells were incubated with secondary antibody for 45 min and washed three times for 5 min with PBS. The fluorescence signal was analyzed with an Accuri C6 Plus flow cytometer (BD Biosciences).

### Experimental infection of pigs with chimeric viruses.

To assess the virulence of chimeric viruses relative to the Shimen strain, 5-week-old healthy pigs were randomly divided into 4 groups (groups 1 to 3, *n *= 3; group 4, *n *= 2), and each group was housed in an individual room. Group 1 was inoculated i.m. with vSM-HCLVE^rns^E2^DomainI^, group 2 was inoculated i.m. with vSM-E2^L108P-I109T^-HCLVE^rns^, group 3 was inoculated i.m. with vSM-HCLVE^rns^E2^DomainII^, and group 4 was inoculated i.m. with the Shimen strain. The inoculation dose of the virus for each group was 10^5^ TCID_50_ (17). Clinical signs and rectal temperature were monitored daily, and anticoagulated blood samples of pigs were collected every 3 or 4 days. CSFV RNA was determined in anticoagulated blood samples by RT-qPCR. The chimeric viruses in the blood samples were titrated in SK6 cells using IFA.

### Pathological examinations.

Macroscopic and microscopic pathological changes of the pig tissues were examined as described previously (50). The tonsils, lymph nodes, kidneys, bladders, and spleens were fixed with 10% formalin and then embedded in paraffin wax. For histopathological examinations, prepared tissue sections were stained with hematoxylin and eosin (H&E).

### Immunohistochemistry.

The spleens of rabbits inoculated with the Shimen strain, C-strain, vSME2^L108P-I109T^-HCLVE^rns^, vSM-HCLVE^rns^E2^DomainI^, or vSM-HCLVE^rns^E2^DomainII^ were subjected to immunohistochemistry examinations using an anti-CSFV E2 antibody as described previously (51).

### Animal ethics.

All animal experiments were carried out in strict accordance with the recommendations in the Guide for the Care and Use of Laboratory Animals of Heilongjiang Province of the People's Republic of China. The protocols were approved by the Committee on the Ethics of Animal Experiments of Harbin Veterinary Research Institute (HVRI) of the Chinese Academy of Agricultural Sciences (CAAS) (approval numbers SY-2018-Ra-002-01, SY-2018-Ra-02, and SY-2019-RA-004 for rabbit experiments and SY-2019-SW-033 for pig experiments).

### Statistical analysis.

SPSS 22.0 software was used to analyze all data. An unadjusted *P* value of <0.05 was considered significant.
